# Lifetime enhancement in QDLEDs via an electron-blocking hole transport layer

**DOI:** 10.1038/s41598-023-45907-5

**Published:** 2023-10-31

**Authors:** Fatemeh Samaeifar, Mohsen Azadinia, Hany Aziz

**Affiliations:** Department of Electrical and Computer Engineering and Waterloo Institute for Nanotechnology, University of Waterloo, 200 University Avenue West, Waterloo, ON N2L 3G1 Canada

**Keywords:** Optics and photonics, Applied physics, Electronics, photonics and device physics, Optical physics, Chemical engineering, Electrical and electronic engineering, Nanoscale materials, Materials for devices, Materials for optics, Nanoscale materials

## Abstract

This study investigates the impact of an engineered hole transport layer (HTL) on the stability of electroluminescent quantum dot light-emitting devices (QDLEDs). The 9-Phenyl-3,6-bis(9-phenyl-9Hcarbazol-3-yl)-9H-carbazole (Tris-PCz) HTL, which possesses a shallower lowest unoccupied molecular orbital (LUMO) energy level compared to the widely used 4,4′-bis(N-carbazolyl)-1,1′-biphenyl (CBP) HTL, is employed to confine electron overflow toward the HTL. Utilizing the Tris-PCz HTL results in a 20× improvement in the electroluminescence half-life (LT50) of QDLEDs compared with conventional QDLEDs using the CBP HTL. Electric and optoelectronic analyses reveal that the migration of excess electrons toward the HTL is impeded by the up-shifted LUMO level of Tris-PCz, contributing to prolonged operational device stability. Furthermore, the augmented electric field at the QD/Tris-PCz interface, due to accumulated electrons, expedites hole injection rates, leading to better charge injection balance and the confinement of the exciton recombination zone within the QD and thus the device stability enhancement. This study highlights the significant influence of the HTL on QDLED stability and represents one of the longest LT50 for a QDLED based on the conventional core/shell QD structure.

## Introduction

Colloidal quantum dots (QDs) possess a unique array of properties that include size-tunable luminescence wavelength, narrow luminescence spectra (full-width at half maximum < 20 nm), high photoluminescence (PL) quantum yield, and amenability to solution-processing. These attributes render them highly attractive for the development of high-performance and low-cost QD-based light-emitting devices (LEDs) (QDLEDs), intended for the next generation of flat panel displays^1–13^. Since their first demonstration in 1994^14^, the electroluminescence (EL) performance of QDLEDs has undergone substantial progress, with their external quantum efficiency (EQE) now reaching the theoretical limit (20%) and their brightness exceeding 10^5^ cd/m^2^^15,16^. Despite the remarkable progress in improving efficiency and brightness, the EL stability of QDLEDs remains relatively low, and the origin of this phenomenon is still not well understood^8,17–21^.

Amidst various contributing factors, the charge injection imbalance into QDs and Auger quenching in them are suggested to be leading culprits behind the limited EL stability of these devices^18,19,22^. While early studies for addressing this issue have focused primarily on improving the chemical and morphological structure of the QDs themselves^23–26^, recent findings show that the charge transport layers also play a major role^12,26,27^. In general, in red QDLED, the energy offset between the highest occupied molecular orbital (HOMO) of the organic hole transport layer (HTL) and the valence band edge energy level of QDs is larger than the energy offset between the conduction band energy level of the inorganic electron transport layer (ETL) and QDs^18,22,23,28^. The easier injection of electrons over holes results in the accumulation of unrecombined residual electrons in QDs and their migration towards the HTL^18,22^, which can induce chemical and morphological changes in the organic HTL^18^.

Among the organic HTLs, 4,4′-bis(N-carbazolyl)-1,1′-biphenyl (CBP) is widely utilized in QDLEDs because of its relatively high hole mobility (2 × 10^−3^ cm^2^/(V s)), which can be used together with inorganic electron transport layers (ZnO/ZnMgO) that also have high electron mobility, leading to improved performance^18,29–33^. However, the long-term stability of such QDLEDs may be a concern due to shallow lowest unoccupied molecular orbital (LUMO) energy level of CBP (− 2.9 eV)^34^, and its susceptibility to morphological changes in the presence of excess electrons^35^. Therefore, there is a need to explore alternative HTLs to achieve both efficient and stable QDLEDs.

In this study, we introduce and investigate the utilization of 9-Phenyl-3,6-bis(9-phenyl-9Hcarbazol-3-yl)-9H-carbazole (Tris-PCz) as an HTL material in QDLEDs. The material has a shallower LUMO energy level (− 2.1 eV) compared to CBP, thereby effectively mitigating the undesired electron leakage and flow into the HTL. The use of Tris-PCz as an organic HTL is explored in inverted red QDLEDs and compared with devices fabricated under the same conditions with CBP HTL. The results demonstrate that by replacing CBP with Tris-PCz, a device EL half-life (LT50), defined as the time for luminance to reach 50% of initial luminescence (L_0_) under continuous electrical driving, of 161,000 h (from an L_0_ of 100 cd m^−2^) for red QDLEDs using a conventional core/shell QD emitter is achieved, 20× longer than QDLEDs with CBP HTL. Further studies indicate not only that the migration of excess electrons into the HTL is blocked by the up-shifted LUMO level of Tris-PCz, but also that confining the electrons within the QD layer accelerates the hole injection rate. This leads to an enhanced charge injection balance and the confinement of the exciton recombination zone within the QD layer, subsequently contributing to higher stability.

## Results and discussion

**Figure 1:**
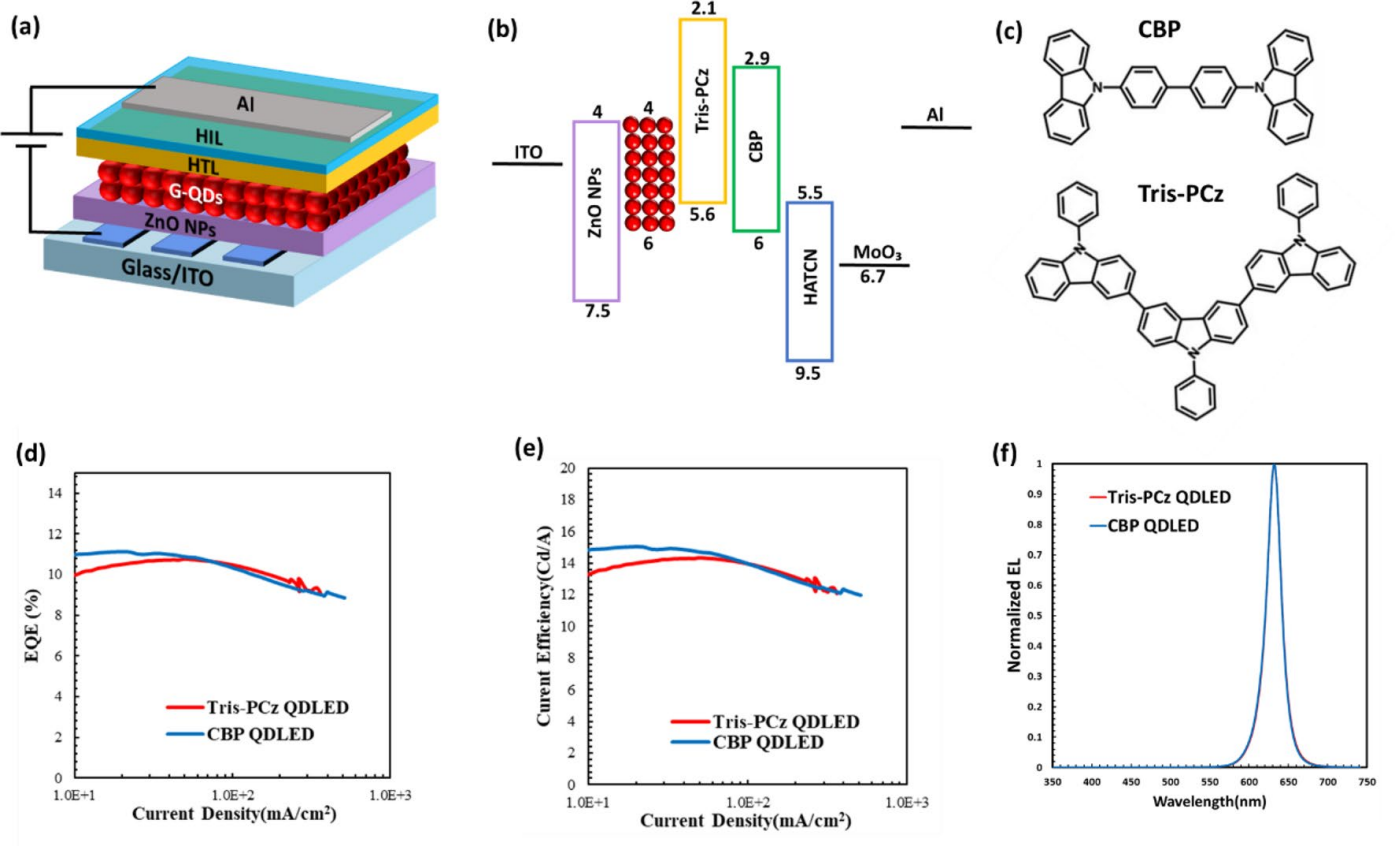
(**a**) Structure, and (**b**) energy band diagram of QDLEDs^37–42^. (**c**) Chemical structures of CBP and Tris-PCz,. (**d**) EQE vs current density characteristics, (**e**) CE vs current density characteristics, and (**f**) normalized EL spectra of the CBP and Tris-PCz QDLEDs. The luminescence is measured while driving the QDLEDs at a 20 mA cm^−2^ current density.

To investigate the effect of the HTL on QDLED performance, inverted red emitting devices were fabricated with the following structure: ITO/ZnO NPs/QD/HTL/hole injection layer (HIL)/Al. In this structure, ITO functions as the electron injection electrode, ZnO NPs act as the ETL, and Al acts as the hole injection electrode. Two groups of QDLEDs of this general structure but with different HTLs/HILs were fabricated: CBP/MoO_3_ (control device, denoted as a CBP QDLED) and Tris-PCz/1,4,5,8,9,11-hexaazatriphenylenehexacarbonitrile (HATCN) (denoted as a Tris-PCz QDLED). The MoO_3_ HIL is commonly used in organic and QDLEDs because of its very deep Fermi level, allowing for better hole injection into HTLs with deep HOMO energy levels, such as CBP^36^. In contrast, the shallower HOMO level of Tris-PCz requires the use of an alternate HIL, such as HATCN, to avoid a large hole injection barrier at the HTL/HIL interface. The device structure and its energy level diagram are depicted in Fig. 1a and b^37–42^, respectively, while the molecular structures of Tris-PCz and CBP are illustrated in Fig. 1c.

The EQE vs current density characteristics of the QDLEDs are depicted in Fig. 1d. Both devices exhibit comparable efficiencies, with maximum EQE values of 10.8% and 11.1% for the Tris-PCz and CBP devices, respectively. In addition, the Tris-PCz and CBP devices show peak current efficiency (CE) of 14.6 and 15 cd/A^−1^, respectively, as displayed in Fig. 1e. The EL spectra of the devices are also shown in Fig. 1f. Both devices show strong QD emission at 630 nm, without any discernible parasitic emission from the other layers, indicating that the majority of radiative recombination occurs within the QD layer in both devices.

**Figure 2:**
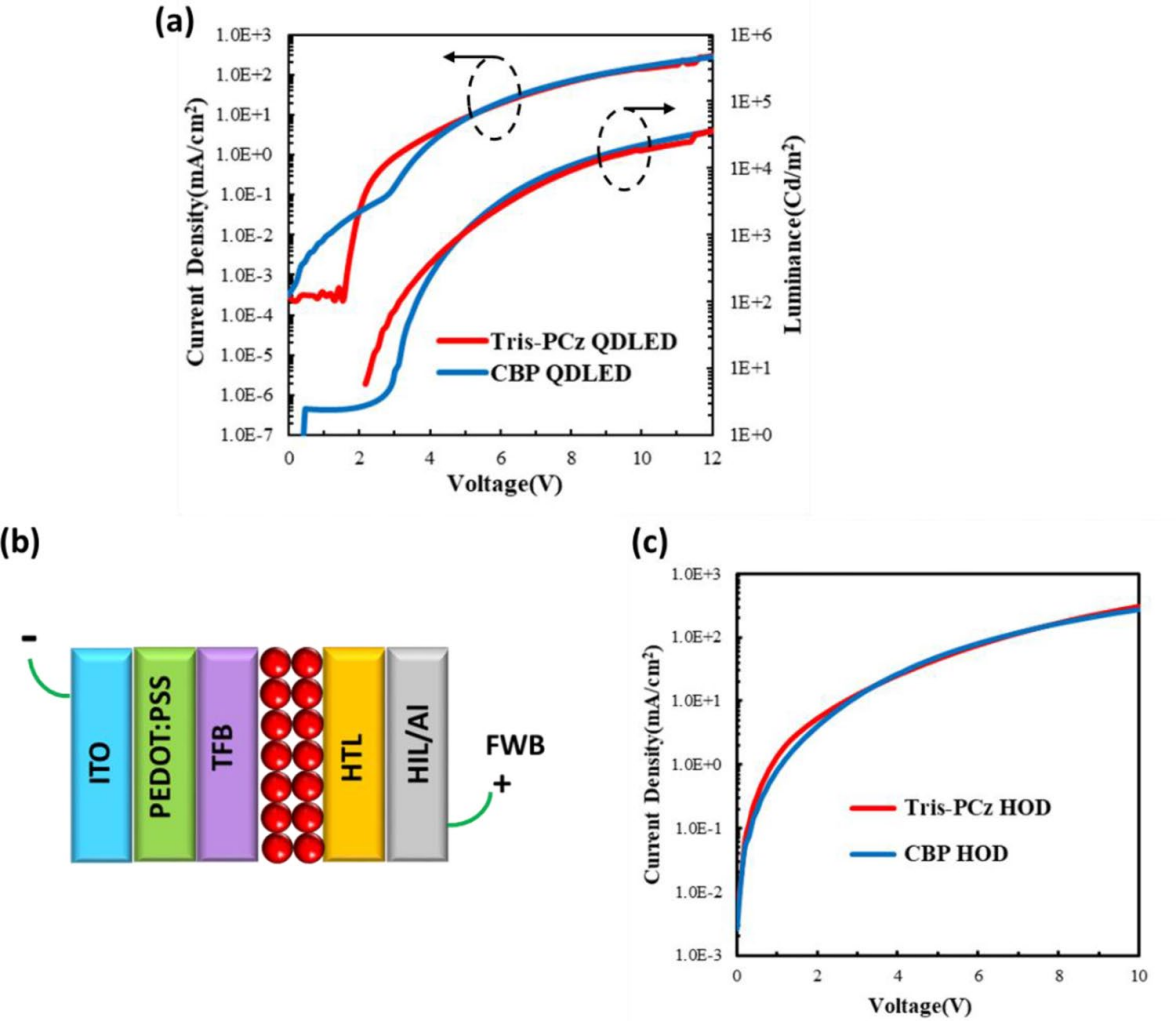
(**a**) Current density and luminance vs voltage characteristics of CBP and Tris-PCz QDLEDs. (**b**) Schematic diagram of the HOD structure. (**c**) J-V characteristics of HODs with CBP and Tris-PCz HTLs.

The Current–voltage–luminance (J-V-L) characteristics in Fig. 2a indicate that the Tris-PCz QDLED exhibits the lower turn-on and reduced leakage current compared to the CBP QDLED. In order to gain an understanding of the underlying reasons and to investigate whether hole injection from the HTL into the QDs is a contributing factor, we also fabricated two groups of hole-only devices (HODs) with the structure of ITO/poly(3,4-ethylenedioxythiophene) polystyrene sulfonate (PEDOT:PSS)/Poly(9,9-dioctylfluorene-alt-N-(4-s-butylphenyl)-diphenylamine) (TFB)/QDs/HTL/HIL/Al, as shown in Fig. 2b. The HODs had the same structure as the QDLEDs except that the ZnO layer was replaced with PEDOT:PSS/TFB layers in order to block electron injection from the ITO cathode into the QD layer. As before, CBP/MoO_3_ were used as the HTL/HIL in the first group, and in the second group, Tris-PCz/HATCN were used instead. Under forward bias (i.e., Al is positively biased relative to ITO), comparable hole currents are observed in both HODs (Fig. 2c), indicating that hole injection and transport are very similar in both devices.

Therefore, the lower turn-on voltage and reduced leakage current observed in Tris-PCz QDLEDs can be attributed to the effective electron-blocking capability of Tris-PCz. QDs carry a negative charge due to the easier injection of electrons compared to holes^43^, and the electron-blocking property of Tris-PCz can lead to the enhancement of confinement-induced Coulomb interactions. Consequently, this facilitates accelerated hole injection while inhibiting excessive electron injection, resulting in the observed lower turn-on voltage and reduced leakage current.

**Figure 3:**
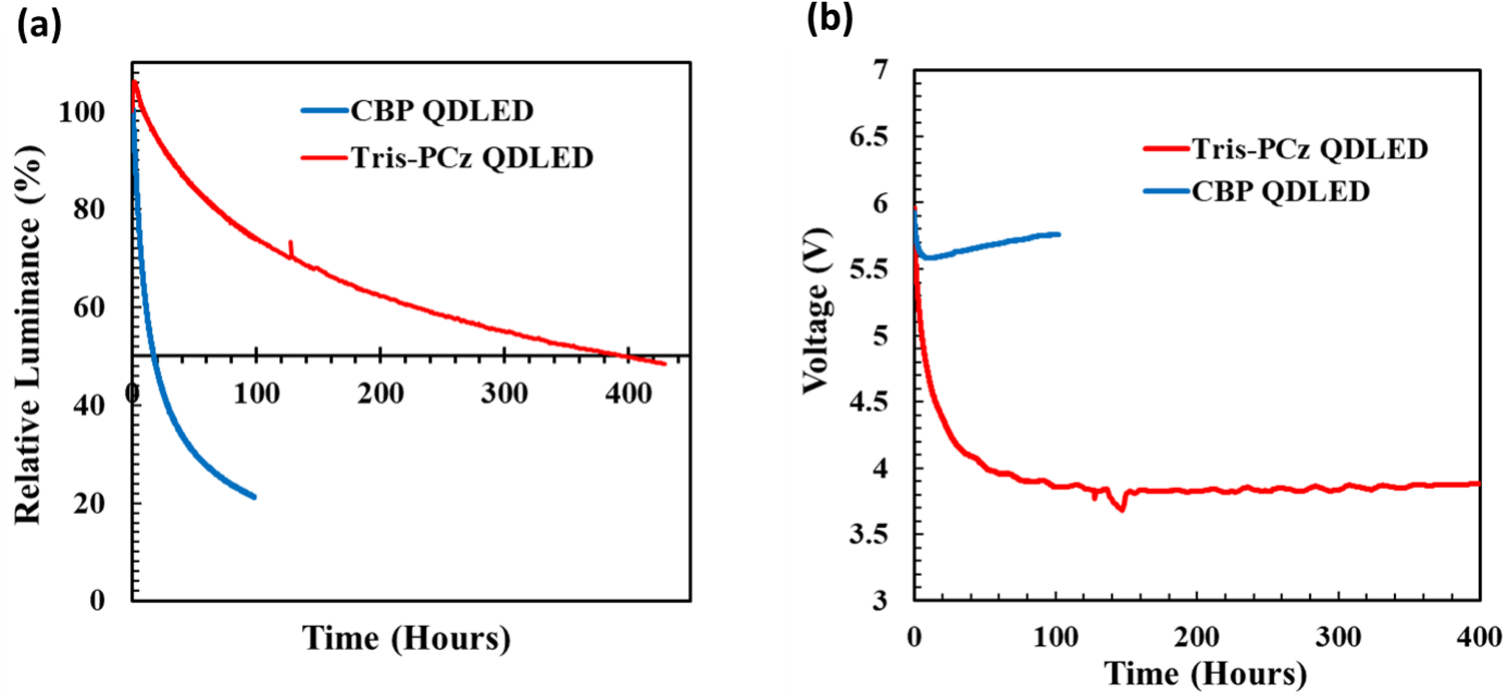
(**a**) Normalized luminance vs time and (**b**) driving voltage vs time of CBP and Tris-PCz QDLEDs measured while driving the QDLEDs at a 20 mA cm^−2^ current density. (**c**) Schematic diagram of the HOD structure. (**d**) J-V characteristics of HODs with CBP and Tris-PCz HTLs.

Figure 3a and b depict the changes in luminance and driving voltage, respectively, for the same devices over time under continuous electrical driving at 20 mA cm^−2^. The Tris-PCz QDLED exhibits a LT50 of 400 h (for a L_0_ of 2800 cd m^−2^). This corresponds to a LT50 of 161,000 h at a L_0_ of 100 cd m^−2^ employing the widely used stability scaling relationship of L_0_^n^LT50 = constant, where n is the acceleration factor of 1.8 frequently utilized for QDLEDs^15,44,45^. The LT50 of the Tris-PCz QDLED is 20× longer than the 20 h LT50 of the CBP QDLED (for a L_0_ of 3000 cd m^−2^), which corresponds to a LT50 of 9000 h for a L_0_ of 100 cd m^−2^. Clearly, these results demonstrate that replacing CBP with Tris-PCz significantly enhances device EL stability.

Figure 3b demonstrates that the driving voltage for both devices falls from its initial value over time in the earlier stages of electrical stress. The voltage drop is, however, notably more pronounced in the Tris-PCz QDLED, where the voltage falls by 2.2 V from its initial value instead of only 0.4 V in the CBP QDLED. In red QDLEDs, due to the large energy barrier between the valence band of the QDs and the HOMO energy level of the HTL, the injection of electrons into the QDs is easier relative to hole injection, leading to charge injection imbalance into the QDs^45^. The presence of a higher concentration of electrons on the QDs at the QD/HTL interface in the case of the Tris-PCz QDLED, due to the shallower LUMO of Tris-PCz and its stronger electron blockage capacity, may promote the hole injection from the HTL into the QDs, leading to a significant reduction in the driving voltage^18^.

After this initial reduction, the driving voltage starts to increase quickly in the case of the CBP QDLED, but it increases only marginally in the case of the Tris-PCz QDLED, as shown in Fig. 3b. The penetration of electrons into organic HTLs is known to cause chemical and morphological changes in them, leading to non-radiative recombination^18,22,46–48^. In the CBP QDLED, electrons are capable of being injected and transported into the HTL, which could result in permanent material degradation, possibly causing the observed increase in the driving voltage.

**Figure 4.**
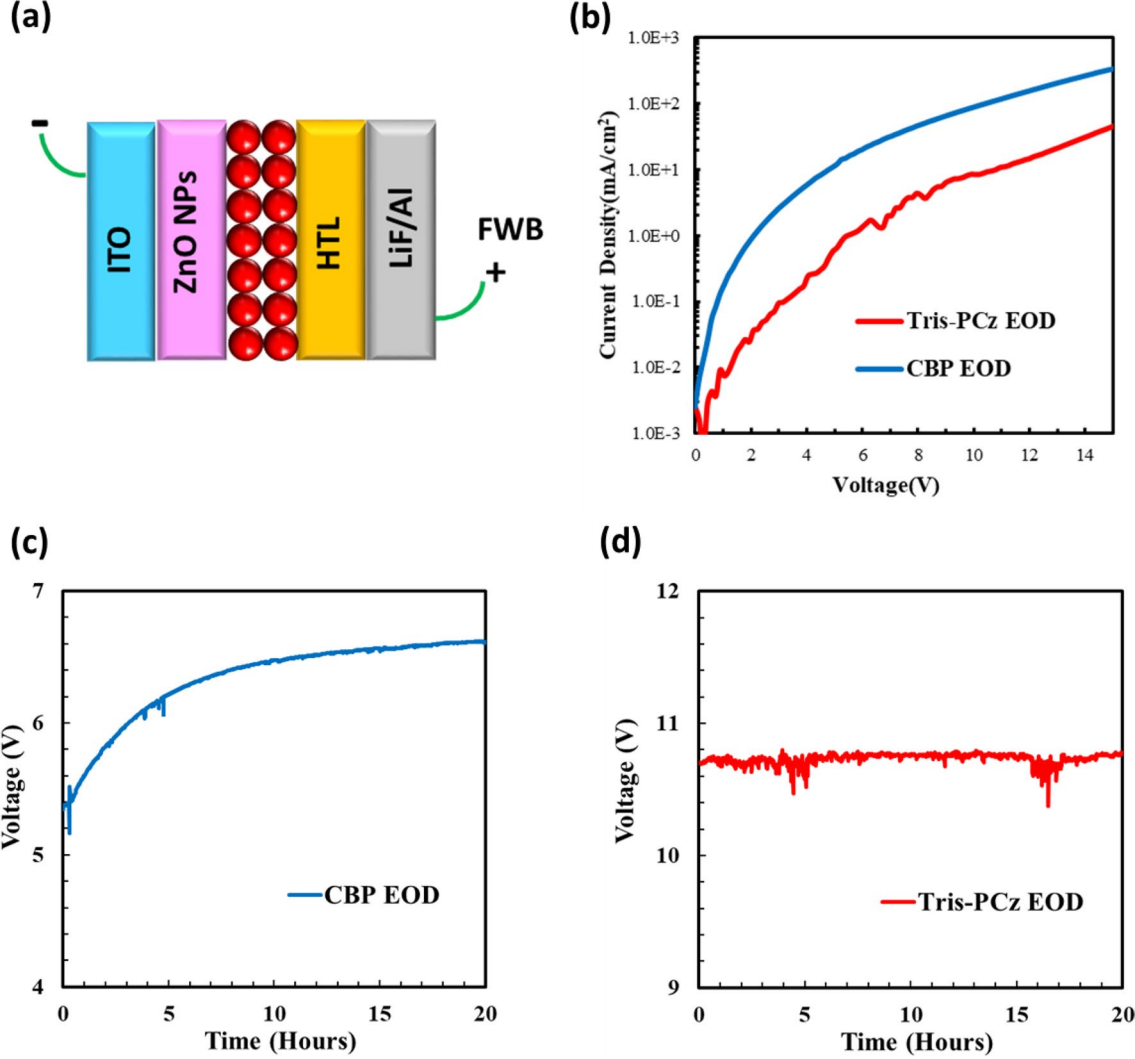
(**a**) Schematic diagram of the EOD structure. (**b**) J–V characteristics of EODs with CBP and Tris-PCz HTLs. Change in driving voltage vs time characteristics for (**c**) CBP, and (**d**) Tris-PCz EODs under constant electrical driving at a 20 mA cm^−2^ current density.

To assess the electron-blocking property by the energy up-shift of the LUMO, we fabricated two groups of electron-only devices (EODs) with the structure: ITO/ZnO NPs/QDs/HTL/LiF/Al, as illustrated in Fig. 4a. CBP and Tris-PCz were used for the HTL in the first and second groups, respectively. Under forward bias, the injection of holes from Al is blocked by the presence of the LiF layer, and therefore the flow of current occurs almost exclusively via the flow of electrons that get injected from the ITO contact and collected at the Al contact. Since the electron injection barriers at the interfaces in-between ITO, ZnO NPs, and QDs are identical to both devices, and the contact barrier between HTL and LiF/Al is insignificant, the measured conductance directly reflects the electron transport property at the interface between the QDs and HTLs. Figure 4b depicts and compares the J–V characteristics of the fabricated EODs. The EOD employing Tris-PCz HTL shows one order-of-magnitude reduction in electron currents compared with the CBP case, which indicates that replacing CBP by Tris-PCz indeed reduces electron transport across the HTL, possibly due to the enlarged energy barrier for electron injection at the QD/HTL interface.

To help glean some insights into the effect of electrons on the HTLs and in turn the stability of the QDLEDs, electrical aging measurements of the CBP and Tris-PCz EODs were carried out by measuring the increase in driving voltage over time when driven at a 20 mA cm^−2^ current density. The results are presented in Fig. 4c and d, respectively. Interestingly, the trends in driving voltage versus time for the EODs mirror those observed in the late stages of stress for the QDLEDs, that are presented in Fig. 3b. The increase in voltage in both the CBP QDLED and the corresponding EOD suggests that the behavior is associated with the flow of electrons (i.e. electron leakage) into the CBP. The presence of electrons in organic HTLs is known to induce structural deformations within organic materials, leading to the formation of non-radiative recombination centers in HTLs^18,22,46–48^. In the case of CBP QDLED, exocyclic C–N bond in CBP is recognized as a weak bond that is susceptible to structural deformations, resulting in the creation of deep trap sites^46,49^. The electrons that escape from the QD layer and migrate into the HTL play a pivotal role in the degradation processes of the HTL. Notably, the increase in voltage resulting from the initial HTL degradation, along with subsequent heat generation, accelerates the leakage of electrons into the HTLs, thereby expediting HTL degradation^22^.

On the other hand, the effective electron-blocking property of Tris-PCz limits this degradation process, leading to a substantially smaller increase in driving voltage under the same constant electrical driving.

**Figure 5:**
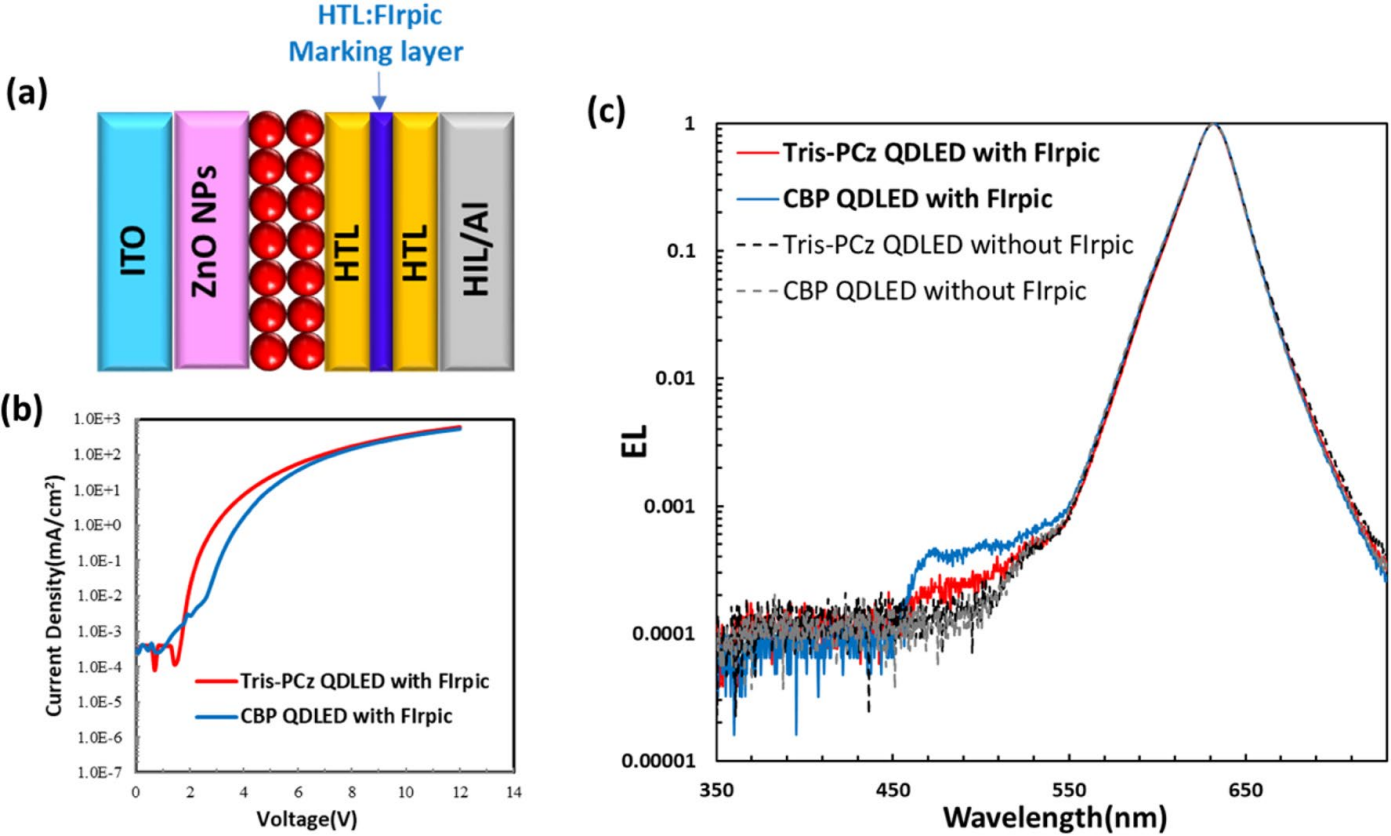
(**a**) Schematic diagram depicting the structure of the QDLEDs with the FIrpic marking layer. (**b**) J–V characteristics, and (**c**) EL spectra from the CBP and Tris-PCz QDLEDs with the marking layer. The spectra from CBP and Tris-PCz QDLEDs without the marking layer are also included for comparison.

To get further insight into the differences in charge distribution between QDLEDs with the Tris-PCz vs the CBP HTLs, a 10 nm thick marking layer of HTL doped with 5% bis[2-(4,6-difluorophenyl)pyridinato-C2,N](picolinato)iridium(III) (FIrpic), a blue organic phosphorescent emitter, was inserted into the HTL, 20 nm away from the QD/HTL interface. FIrpic was chosen as the luminescent dopant for the marking layer because of its emission in the 450–550 nm range, enabling clear differentiation from the red EL of the QDs. The devices with the structure of ITO/ZnO NPs/QD/HTL (20 nm)/HTL:FIrpic (10 nm)/HTL (20 nm)/MoO_3_ (5 nm)/Al (100 nm) were fabricated, as depicted in Fig. 5a. Figure 5b shows the J–V characteristics of fabricated devices with the marking layer, which are very similar to J–V characteristics of the QDLEDs without the marking layer, as observed in Fig. 2a, indicating that the marking layer indeed perturbs charge distribution in the devices only minimally. Figure 5c shows the EL spectra recorded from the QDLEDs incorporating different HTLs while driven at 20 mA cm^−2^ current density. The spectra of the Tris-PCz and CBP QDLEDs without the marking layer are also included for comparison. All spectra are normalized to the QD emission band peak intensity to facilitate comparison. The CBP device shows significant emission from FIrpic, indicating that a considerable number of electrons manage to penetrate the HTL and reach the marking layer, where they recombine with nearby holes to produce EL. In contrast, the spectrum of the Tris-PCz device displays only very weak (although still discernible) emission from FIrpic, suggesting that the penetration of electrons into the HTL is much less in comparison.

These results show the enhanced stability of the Tris-PCz QDLEDs is possibly associated with a lower concentration of electrons in the HTL and improved confinement of the exciton recombination zone within the QD layer, consistent with the above results. The presence of electrons in high concentrations in the HTL could accelerate HTL degradation and exacerbate the charge injection imbalance into the QDs, potentially leading to decreased device efficiency during operation^18^.

**Figure 6:**
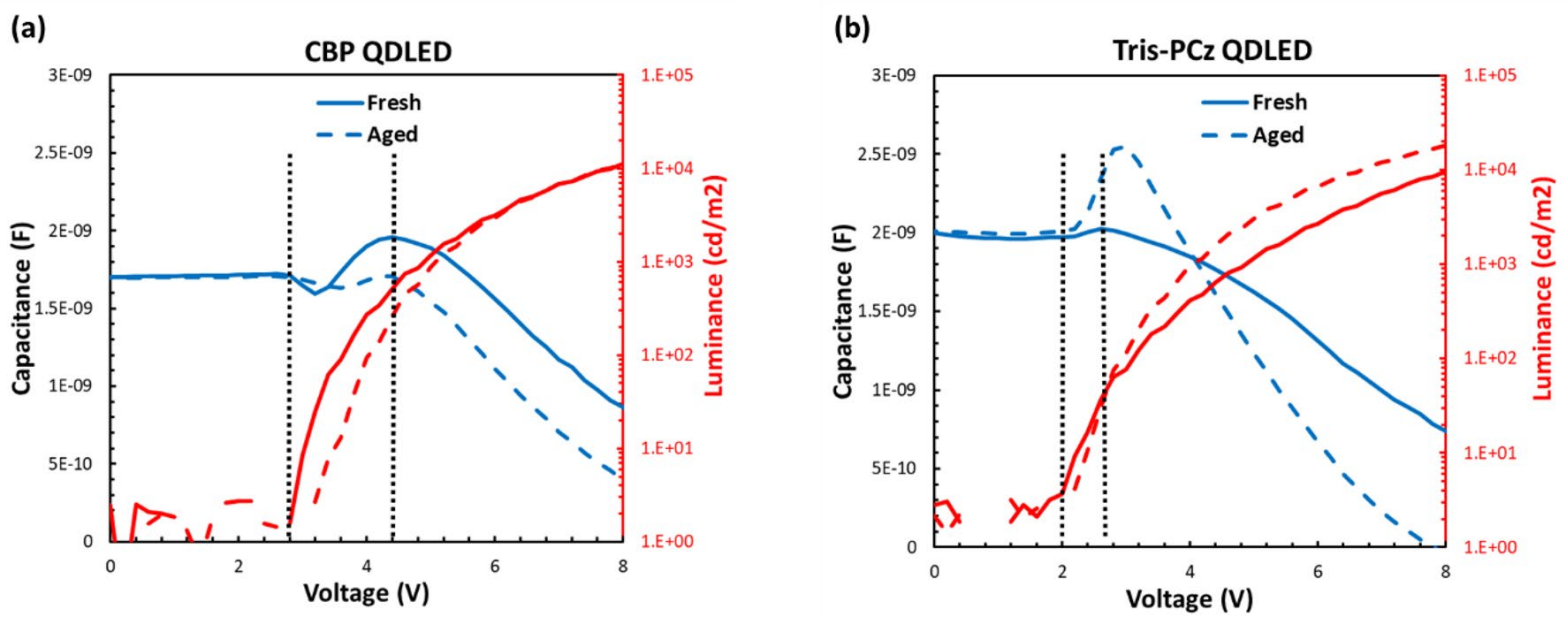
The capacitance–voltage(C-V) and luminance-voltage (L-V) characteristics of fresh and aged QDLEDs using (**a**) CBP, and (**b**) Tris-PCz.

We also compared the capacitance–voltage–luminance (C-V-L) characteristics of the Tris-PCz and CBP QDLEDs, for fresh and electrically aged devices, to further verify and elucidate the impact of increased electron concentration in the QDs on the interaction with holes in the QDs and/or at the QDs/HTL interface. Figure 6a and b represent the C-V-L characteristics of the fresh (i.e. unaged) and aged devices, the latter collected after electrical driving at 20 mA cm^–2^ for 100 h, with CBP and Tris-PCz QDLEDs, respectively. Below the turn-on voltage (V_on_) the capacitance reflects the geometric capacitance of the ETL, QD, and HTL depleted layers connected in series. Above V_on_, the capacitance begins to increase, pointing to charge injection and accumulation within the device stack, eventually reaching a peak. Since in a red QDLED device, electron injection is easier than hole injection^50^, the capacitance rise means the HTL is the only layer remaining depleted while the other layers reach a flat band. Indeed, the peak capacitance values of ~ 1.95 nF and ~ 2.0 nF for the fresh CBP and Tris-PCz QDLEDs, respectively, are close to the geometric capacitance of a 50 nm-thick HTL layer (~ 2 nF). The slightly higher peak capacitance of the Tris-PCz QDLED could be attributed to the higher electron concentration at the QD/HTL interface of this device^51^, as was concluded earlier.

The point at which capacitance starts to decrease sharply marks the onset of efficient radiative recombination. As shown in Fig. 6, in the fresh CBP QDLED, V_on_ is 2.4 V, and the peak capacitance is observed at a much higher voltage, 4.4 V, whereas in the fresh Tris-PCz QDLED, V_on_ is 2.1 V, and the peak capacitance is more quickly reached, at 2.5 V. The occurrence of peak capacitance at a much higher voltage than what is needed for radiative recombination is an indicator of charge imbalance, and may be a leading factor to the lower stability of CBP QDLEDs^52^.

At higher voltages, the capacitance of both fresh QDLEDs decreases, indicating the annihilation of charges through the recombination process. Interestingly, the capacitance decreases much faster and to a lower capacitance value in the case of the Tris-PCz QDLED, pointing to a faster removal of electrons at the QD/HTL interface. It is, therefore, quite possible that enriching the QD/HTL interface with electrons, as a result of the up-shifted LUMO level of Tris-PCz, facilitates hole injection across the HTL/QD interface, thus accelerating the annihilation of charges at the interface. The CBP QDLED, in contrast, exhibits a slower charge annihilation process.

The C-V-L characteristics of the aged Tris-PCz QDLED also reach their peak capacitance at a lower voltage, indicating that charge balance remains better compared to the CBP QDLED even after aging. Also notably, the peak capacitance decreases (from ~ 1.95 to ~ 1.7 nF) after aging in the CBP QDLED, whereas it increases (from ~ 2 to ~ 2.5 nF) in case of the Tris-PCz device. The increase in peak capacitance after aging can be ascribed to the increase in electron concentrations over time at the QD/HTL. In this context, the decrease in peak capacitance for the CBP device may be indicative of an increase in electron leakage, leading to a reduced accumulation of electrons at the QD/HTL interface. The presence of high concentration of electrons in CBP can cause considerable degradation in the HTL’s hole injection/transport properties^53^, which, in turn, can lead to device degradation. Moreover, the observation that the capacitance decreases to almost zero in the aged Tris-PCz QDLED is consistent with a scenario where the high number of electrons at the QD/HTL interface expedites hole injection across the HTL/QD interface. This, in turn, leads to an improved charge balance, facilitating faster removal of charges and hence enhancing the stability of the devices.

## Conclusion

In this work, we investigated the impact of an engineered HTL on the EL stability of QDLEDs. Tris-PCz HTL, which has a shallow LUMO energy level, was utilized to confine electron overflow toward the HTL and to prevent HTL degradation caused by electrons. Inverted red QDLEDs were fabricated using the Tris-PCz HTL and were compared with devices fabricated under the same conditions using CBP HTL. The results demonstrate that by replacing CBP with Tris-PCz, an LT50 of 161,000 h (at a luminescence of 100 cd m^−2^) for red QDLEDs using a conventional core/shell QD emitter is achieved, 20× longer than QDLEDs with CBP HTL. The electrical and optoelectronic studies indicate not only that the migration of excess electrons is blocked by the up-shifted LUMO level of Tris-PCz, but also that the presence of accumulated electrons expedites the hole injection rate. This leads to a better charge injection balance as well as effective confinement of the exciton recombination zone within the QD layer, subsequently enhancing device stability. This work sheds light on the importance of HTL engineering in QDLEDs stability and also holds the promise of realizing stable QDLEDs for use in applications that require high stability.

## Methods

### QDLED fabrication

The QDLEDs were fabricated on 100 nm thick ITO-patterned glass substrates (Kintec) with a sheet resistance of 20 Ω sq^−1^. The ITO substrates were cleaned using Micro 90 (Cole-Parmer), and subsequently sonicated with deionized water, acetone, and isopropanol solutions in sequence. Following the cleaning process, the substrates underwent a 5-min oxygen plasma treatment to enhance surface wettability. 30 mg mL^−1^ ZnO NPs dispersed solution (SkySpring Nanomaterials, Inc.), filtered two times through a 0.22 μm polypropylene filter, was spin-coated onto the substrates at 3000 rpm, followed by baking at 400 °C on a hotplate for 30 min. 12.5 mg mL^−1^ red CdSe/ZnS QDs (Mesolight Inc.) suspended in octane, with a peak EL emission wavelength of 630 nm and a 75% PL quantum yield (PLQY), were spin-coated at a 3000 rpm and then annealed at 50 °C for 30 min. Subsequently, CBP (Angstrom Engineering), Tris-PCz (Luminescence Technology), HATCN (Angstrom Engineering), MoO_3_ (Angstrom Engineering), and Al (Angstrom Engineering) with thicknesses of 50 nm, 50 nm, 10 nm, 5 nm, 100 nm, respectively, were deposited at rates of 0.1–2 Å s^−1^ in an Angstrom Engineering EvoVac thermal evaporation chamber at a base pressure of 5 × 10^−6^ Torr. All deposition processes were performed in a nitrogen-filled glove box and vacuum chamber.

### QDLED characterization

The luminance of the QDLEDs was measured using a Minolta Chroma Meter CS-100, and current–voltage–luminance measurements were conducted with an Agilent 4155C semiconductor parameter analyzer connected to a silicon photodiode. Spectral measurements of the QDLEDs were obtained using an Ocean Optics QE65000 spectrometer. EL lifetime measurements were carried out in an M6000PLUS OLED Lifetime Test System, maintaining a constant driving current at 20 mA cm^−2^. Capacitance–voltage characteristics were measured using a Keithley 4200 semiconductor analyzer with a capacitance–voltage unit (4215-CVU). The capacitance was measured using an AC voltage with a 20 mV room-mean-square amplitude and a frequency of 10 kHz.

## Data Availability

The data that supports the findings of this study are available from the corresponding author upon reasonable request.
